# Molecular Features Related to HIV Integrase Inhibition Obtained from Structure- and Ligand-Based Approaches

**DOI:** 10.1371/journal.pone.0081301

**Published:** 2014-01-08

**Authors:** Luciana L. de Carvalho, Vinícius G. Maltarollo, Emmanuela Ferreira de Lima, Karen C. Weber, Kathia M. Honorio, Albérico B. F. da Silva

**Affiliations:** 1 Instituto de Química de São Carlos, Universidade de São Paulo, São Carlos, Brazil; 2 Centro de Ciências Naturais e Humanas, Universidade Federal do ABC, Santo André, Brazil; 3 Escola de Artes, Ciências e Humanidades, Universidade de São Paulo, São Paulo, Brazil; 4 Centro de Ciências Exatas e da Natureza, Universidade Federal da Paraíba, João Pessoa, Brazil; University of Cambridge, United Kingdom

## Abstract

Among several biological targets to treat AIDS, HIV integrase is a promising enzyme that can be employed to develop new anti-HIV agents. The aim of this work is to propose a mechanistic interpretation of HIV-1 integrase inhibition and to rationalize the molecular features related to the binding affinity of studied ligands. A set of 79 HIV-1 integrase inhibitors and its relationship with biological activity are investigated employing 2D and 3D QSAR models, docking analysis and DFT studies. Analyses of docking poses and frontier molecular orbitals revealed important features on the main ligand-receptor interactions. 2D and 3D models presenting good internal consistency, predictive power and stability were obtained in all cases. Significant correlation coefficients (*r^2^* = 0.908 and *q^2^* = 0.643 for 2D model; *r^2^* = 0.904 and *q^2^* = 0.719 for 3D model) were obtained, indicating the potential of these models for untested compounds. The generated holograms and contribution maps revealed important molecular requirements to HIV-1 IN inhibition and several evidences for molecular modifications. The final models along with information resulting from molecular orbitals, 2D contribution and 3D contour maps should be useful in the design of new inhibitors with increased potency and selectivity within the chemical diversity of the data.

## Introduction

According to the latest UNAIDS (Joint United Nations Program on HIV/AIDS) global report, 34 million people were infected with HIV at the end of 2011. Although the number of newly infected people worldwide has been decreasing by 20% since 2001, in some parts of the world as Middle East and North Africa this number has increased by more than 35% [1]. Mainly due to antiretroviral therapy, the number of people dying from AIDS-related causes is also decreasing worldwide, with an estimated 24% decline since 2005. Despite progress in HIV antiretroviral therapy, some improvements are still needed such as the development of drugs able to tackle wild-type and mutant viruses without viral breakthrough and also with better bioavailability and elimination half-life in order to allow once-a-day doses and consequently a better retention on treatment [1]–[3].

Among the molecular targets of existing antiviral drugs that can be explored to design improved new drugs, HIV integrase (HIV-1 IN) is still a noteworthy target due to its pivotal role in the integration of the viral genome into host cell chromatin, which is a fundamental step in the replication cycle of HIV. HIV-1 IN catalyzes the integration of viral DNA into the host DNA in two steps: 3 -processing and strand transfer. The first step, 3-processing, involves a hydrolyzing reaction that removes a terminal dinucleotide to recess a CA-3 terminal. The CA-3 sequence is generally conserved on the third and fourth position from both 3-ends of long terminal repeats of the viral DNA. The second step, called strand transfer, is a transesterification reaction of the recessed 3-ends with the phosphodiester backbone of the host cell DNA. Both ends of the viral DNA are joined to the host DNA at the same time, and subsequently repair of the nick by host cell repair machinery completes the integration sequence [4], [5].

Many advances in the discovery of HIV inhibitors have been achieved in the last decades. Inhibitors derived from 4-aryl-2,4-diketobutanoic acid containing a diketoacid moiety (DKA) were identified in 2000 by Merck [6]. Their antiviral activity in cell culture was attributed to inhibition of strand transfer, which was confirmed by the rapid development of resistant mutations of HIV-1 IN [7]. Because of their high selectivity and inhibition of viral activity in nanomolar range, these compounds have become important chemotherapeutic agents [8]. The first approved HIV-1 IN inhibitor is raltegravir (MK-0518), obtained from the optimization of DKA lead compounds containing hydroxy-8-(1,6)-7-naphthyridine carboxamide and N-alkyl-5-hydroxypyrimidinone groups [9]–[14]. The functionality of the 1,3-β-diketoacid group is essential for inhibitory activity, as confirmed by the crystal structure [15] of IN bound to the prototype DKA, 1-(5-chloroindol-3-yl)-3-hydroxy-3-(2H-tetrazol-5-yl)-propenone (5-CITEP).

Although some QSAR and docking studies have been performed on different series of HIV-1 IN inhibitors over the last few years, the union and rationalization of experimental results with theoretical studies, especially in the case of electronic properties, are still necessary in order to obtain predictive relations that may lead to the design of new inhibitors acting as anti-HIV drugs [16]–[18]. In this work, molecular features related to the biological activity of a series of *N*-alkyl-5-hydroxypyrimidinone derivatives acting as HIV-1 IN inhibitors were investigated by integrating molecular docking, quantum chemistry, 2D and 3D QSAR techniques. With these approaches, we were able to explore the binding modes of these inhibitors with the HIV-1 IN enzyme, as well as to examine the structure-activity relationships for the HIV-1 IN inhibitory activity of these compounds using CoMFA and HQSAR. The QSAR models generated may provide useful information about the design and synthesis of more potent HIV-1 IN inhibitors with predetermined affinity.

## Materials and Methods

### 1. Data set

**Figure 1:**
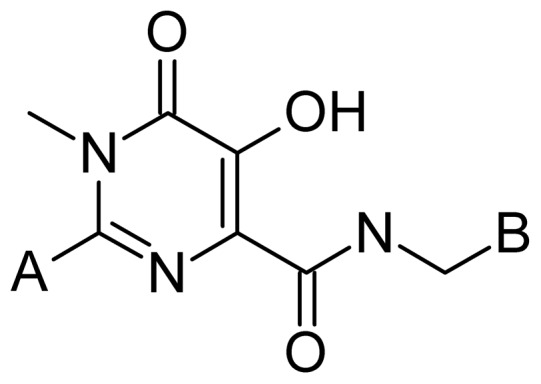
Representative structure of the chemical diversity in the data set.

The series of HIV-1 IN inhibitors employed in the development of 2D and 3D QSAR models is constituted by 79 compounds selected from the literature [10]–[14]. Chemical structures and the value of biological activity (IC_50_) for the complete set of compounds are listed in Table S1. The IC_50_ values vary from 2 to 2000 nM (Table S1) and were measured under the same experimental conditions, which is a fundamental requirement for successful QSAR studies. The IC_50_ values were converted to the corresponding pIC_50_ (−log IC_50_) and used as dependent variables in the QSAR investigations. From the original data set containing 79 inhibitors, 64 (1–64, 80%, Table S1) compounds were selected as members of the training set (model construction) and the other 15 compounds (65–79, 20%, Table S1) as members of the test set (external validation). These compounds were selected carefully in such a way that the structural diversity and the pIC_50_ distribution (cluster analysis) of the data set were well represented in both training and test sets. Figure 1 displays a representative structure of the chemical diversity in the data set.

**Figure 2:**
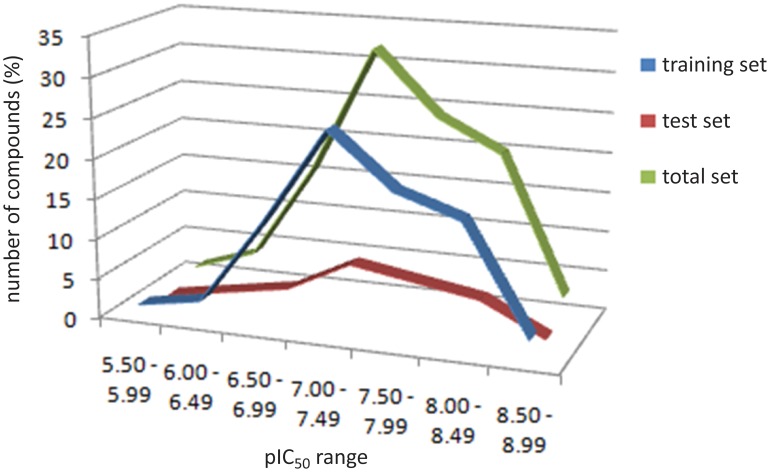
Distribution of pIC_50_ values for training and test sets.

The distribution of pIC_50_ values is shown in Figure 2. This appropriate distribution, in relation to the biological property, represents an important factor when one evaluates the internal and external consistency of the QSAR models. From Figure 2, we can conclude that the set arranged for the HIV-1 IN inhibitors is suitable for the development of predictive 2D and 3D QSAR models in this work.

The inhibitor structures were built using the CONCORD module [19] available in SYBYL 8.1 package [20]. Subsequently, each molecule in the data set was optimized and energetically minimized employing the atom-centered partial charge AM1-ESP calculations, implemented in MOPAC 6.0 [21]. The 3D generated structures were used as starting points for the structural alignment necessary for the CoMFA analyses, which were undertaken through molecular docking. All the calculations and visualizations for QSAR studies were performed using the SYBYL 8.1 package.

### 2. Molecular Docking

Docking simulations of the HIV-1 N inhibitors were performed to study the appropriate binding orientations and conformations and to provide the molecular alignment for the whole data set required for CoMFA studies. The protein structure, determined with a 2.1 Å resolution, in complex with the diketoacid inhibitor 5CITEP and a magnesium atom, was selected from Protein Data Bank (PDB code 1QS4) [15]. GOLD 5.0 [22] was used in the docking simulations of the inhibitors at the enzyme's binding site.

**Figure 3:**
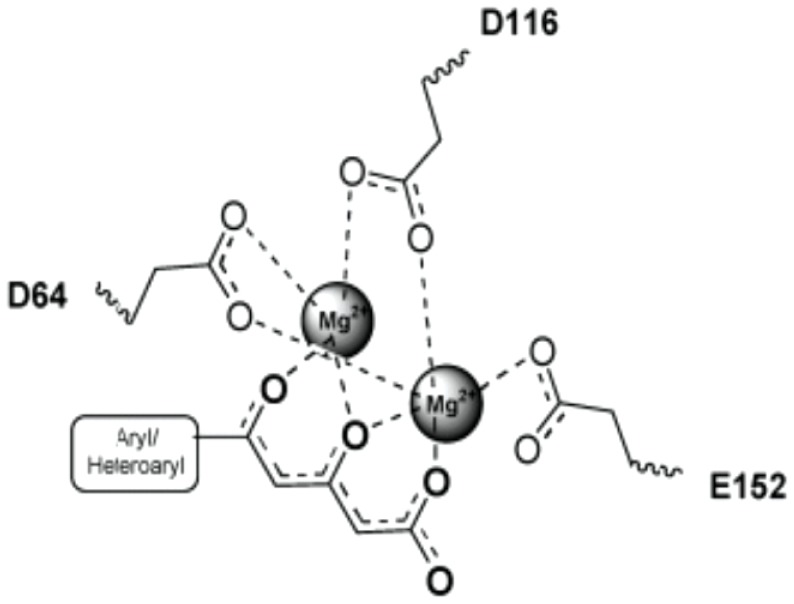
Interactions between HIV-1 IN and inhibitors, as proposed by Goldgur et al. [24].

The structural preparation of the selected target involved the following steps: (i) analysis of the main aminoacids in the active site; (ii) ligand removal of the active site; (iii) adding a second Mg^2+^ ion between D64 and E152 residues, using PyMOL software [23]; (iv) elimination of water molecules and (v) addition of hydrogen atoms by means of GOLD 5.0 software. Furthermore, the interactions between HIV-1 IN and inhibitors, as proposed by Goldgur *et al.* [24], were considered as guides in the molecular dockings (see Figure 3).

Based on the structural analysis of intermolecular interactions responsible for the molecular recognition process, some features were set out in the docking process. A 5 Å cavity was defined involving residues around 5CITEP ligand. Regarding the constraints, each Mg^2+^ ion should be chelated by the oxygen atoms from the hydroxypyrimidinone group and by the oxygen atoms from D116, D64 and E152 residues (catalytic triad DDE). 30 runs of the GOLD search were carried out for each compound in the data set, allowing total bonds, dihedrals and angles flexibility. Ligand poses were ranked using GoldScore function [22].

The docked solutions of all ligands were used in the alignment process. The best 10 rank solutions for each ligand were analyzed. Each conformation was evaluated to satisfy the pharmacophoric hypothesis presented in Figure 3. Thus, it was found that the chemical groups of the inhibitors were correctly placed on the protein active site. The degree of similarity between the conformations of different molecules, as well as their engagement within the binding site, was also evaluated. Based on these criteria, one conformation for each molecule was chosen.

Finally, the chosen conformations for raltegravir, the most and the least potent inhibitors of the data set were submitted to DFT (Density Functional Theory) single point calculations, employing B3LYP functional [25], [26] and 6–31G** basis set [27], by using Gaussian 03 package [28], in order to analyze the localization of frontier molecular orbitals in these molecules and correlate these findings with their affinities to HIV-1 IN.

### 3. CoMFA Analyses

3D QSAR methods are based on the three-dimensional characters of ligand-receptor interactions and in the use of interaction molecular fields as descriptors. In this work, Comparative Molecular Fields Analysis (CoMFA) [29] method was used in order to better understand and explore the electrostatic and steric contributions of HIV integrase inhibitors for its biological activity.

**Figure 4:**
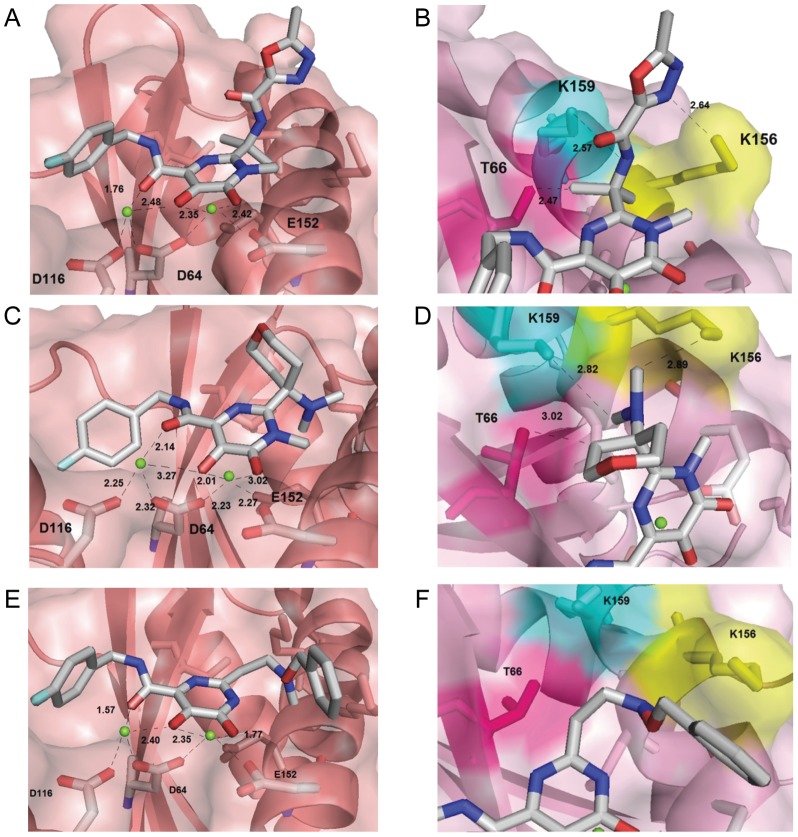
Interactions between Mg^2+^ ions (left) and T66, K159 and K156 residues (right) and raltegravir drug (A and B), the most active (2) (C and D) and the least active (37) molecules from the data set (E and F).

A crucial step in the generation of the CoMFA model is the 3D molecular alignment of the data set [30], [31]. As mentioned before, the 3D alignment used in this study was based on the molecular docking of the inhibitors in the enzyme active site. After the molecular alignment, the steric and electrostatic properties were calculated according to the Lennard-Jones and Coulomb potentials, respectively. For this, the 79 aligned molecules (see Figure 4) were placed in a 3D grid box of 2.0 Å in the x, y, and z directions. Steric and electrostatic fields were generated at each grid point with Tripos force field using a sp^3^ carbon atom probe carrying a +1 net charge. The default value of 30 kcal/mol was set as the maximum steric and electrostatic energy cutoff. 3D models were constructed using Partial Least Square (PLS) technique, with leave-one-out cross validation and a test set (not used in the model building) as external validation test, and also the region-focusing method was employed in order to obtain a model with maximum predictive ability and to increase the resolution of CoMFA models.

### 4. HQSAR

Hologram Quantitative Structure-Activity (HQSAR) methodology requires only 2D structures and the corresponding biological activity as input, allowing the investigation of a wide variety of bioactive compounds. This technique explains differences in the biological activity of a series of molecules by quantifying variations within their molecular holograms and using the PLS method [32].

The main steps involved in HQSAR analyses are: substructural fragmentation for each molecule in the training set; molecular hologram generation; statistical analysis and model generation; test set selection and external validation. To generate the molecular fragments and holograms, some parameters can be varied:

- Fragment distinction including information on A (atoms), B (bonds), C (connectivity), H (hydrogen atoms), Ch (chirality), DA (donors and acceptors of hydrogen bond);
- Hologram Lengths of 53, 69, 61, 71, 83, 97, 151, 199, 257, 307, 353 and 401.
- Fragment size: this parameter controls the minimum and maximum number of atoms in each fragment. Initially, it was used the standard size (4–7 atoms) for the generation of the fragments; then, the size was varied from 2 to 10 until the best model was found.

The particular nature of the sub-structural fragments generated by HQSAR and, consequently, the information contained in the resultant molecular holograms is changed by adjusting these parameters. Finally, the correlation of the obtained descriptors with the biological property was modeled using the PLS method.

## Results and Discussion

### 1. Molecular Docking

From the molecular docking results, it was observed that all the compounds showed a similar interfacial binding mode in which the hydroxypyrymidinone group chelates Mg^2+^ ions together with the catalytic triad DDE (Figures 4A, 4C and 4E), as discussed in the literature [5], [6], [24], [33]. In addition to the interactions with Mg^2+^, there are also interactions with important residues in the integration process, such as T66 and the two lysine residues (K156 and K159) involved in DNA strand viral binding in the strand transfer process catalyzed by integrase [34]. Figures 4B and 4D illustrate these interactions with raltegravir drug and molecules **2** and **37**.

From Figures 4B and 4D, it is possible to observe the interactions of fragment A from raltegravir drug and molecule 2, respectively, with HIV-1 IN residues. There are hydrogen bonds between the oxygen atom from residue T66 and the hydrogen atom from one of the methyl groups, the oxygen from residue K159 and the hydrogen atom from carboxamide group and also between residue K156 and the oxadiazole ring, which are in agreement to the model proposed by Goldgur *et al.*, [15]. It's possible to see that the length of the hydrogen bonds for the raltegravir is smaller in comparison with this length in the most active molecule (**2**). In Figure 4F we can see that, for the least active molecule (**37**), there are no interactions from this portion of the molecule with these residues.

### 2. Frontier molecular orbitals and HIV-1 IN affinity

**Figure 5:**
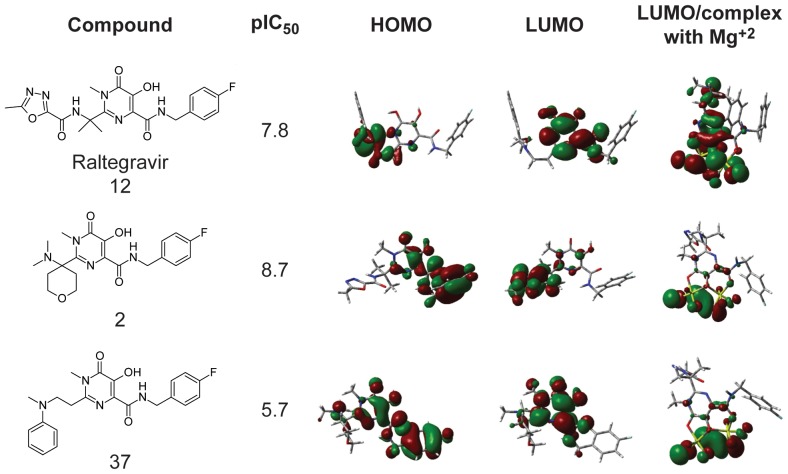
HOMO and LUMO for raltegravir (12) and for the most (2) and least active (37) molecules.

Figure 5 displays the frontier molecular orbitals for raltegravir (**12**), the most (**2**) and the least active ligand (**37**) studied and their complexes with Mg^2+^. As discussed before, the activity of these inhibitors is dependent on the Mg^2+^ ions from the catalytic site of HIV-1 IN, which acts as a bridge between the catalytic triad DDE, the viral cDNA and the host DNA. In order to understand the electronic features of these molecules involved in this interaction, the plots of HOMO and LUMO orbitals of the molecules alone, as well as for the molecules in complex with Mg^2+^ ions, were obtained by DFT/B3LYP/6-31G** calculations.

From Figure 5, it is possible to observe that for the most active molecules (which contain a 8-hydroxy-(1,6)-naphthyridine-7-carboxamide group), HOMO lies in the pharmacophoric portion responsible for the complexation of Mg^2+^ ion, with a higher electronic density on oxygen atoms. Thus, the rings at 8-hydroxy-(1,6)- naphthyridine -7-carboxamide group contribute with a nucleophile character to these molecules, in contrast with the 1,3-diketoacid group, which lacks aromatic rings. Consequently, naphthyridine derivatives are more active at HIV-1 IN than diketoacids. Analyzing the molecules in complex with Mg^2+^, one can see that LUMO is concentrated on Mg^2+^ ions in the most active molecules, since this is the site for putative electronic transfer. In the case of the least active molecule, LUMO spreads over the whole molecule, which can in part accounts for the less affinity of this molecule at HIV-1 IN.

### 3. CoMFA

**Figure 6:**
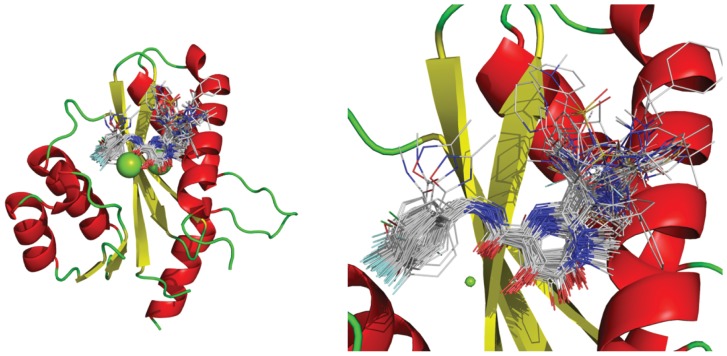
Molecular alignment for the complete data set obtained from docking analyses.

The 79 molecules aligned for the 3D QSAR modeling are shown in Figure 6. The characteristics observed in this model are consistent with the hypothesis of diketoacid binding in complex with the two Mg^2+^ ions and the catalytic triad DDE.

As the first CoMFA model showed unsatisfactory statistical parameters, we have applied the region-focusing procedure. The best statistical results obtained are displayed in Table 1. From Table 1, it can be seen that an initial analysis without using region focusing (an advanced method of noise reduction) produced a low cross-validated correlation coefficient (*q^2^* = 0.401, with four PLS components). Thus, we have applied the region-focusing procedure, which weights the lattice points in a CoMFA region in order to enhance or attenuate their contribution to the PLS analysis, allowing to increase the resolution of CoMFA models. So, our grid points were weighted by standard deviation coefficient values ranging from 0.3 to 1.2, with a grid spacing varying from 0.5 to 1.5 Å. The best statistical results were obtained when region focusing was weighted by a standard deviation coefficient of 1.0 (*r^2^* = 0.904, *q^2^* = 0.719 and six PLS components). Within this CoMFA model, the contributions of steric and electrostatic fields correspond to 49% and 51% of the total variance, respectively.

**Table 1:** Statistical parameters obtained from CoMFA analyses.

Model	*q^2^*	*r^2^*	*N*	*Percentage of contribution*
**no-focusing**	0.401	0.947	4	-
**w = 1.0**	0.719	0.904	6	Steric = 49% Electrostatic = 51%

**Figure 7:**
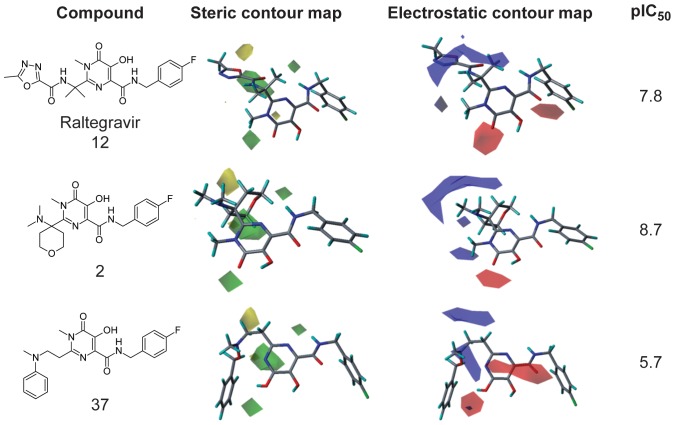
3D contour maps for raltegravir (12), the most (2) and the least active ligand (37). Blue and red polyhedral indicate that substitution by positive and negative groups, respectively, can improve the biological activity. Green and yellow ones represent favorable and unfavorable steric regions, respectively.

From the best CoMFA model obtained, 3D contour maps were generated, i.e. graphical results can be analyzed considering steric and electrostatic fields. Favorable and unfavorable regions for substitution by bulky substituents are represented in green and yellow, respectively. Electrostatic features are characterized in such a way that red contours represent regions in which electronegative substituents may increase the biological activity, whereas blue contours indicate regions in which electropositive groups would contribute to enhance activity. The contour maps for raltegravir (**12**), the most active (**2**) and the least active (**37**) molecules are shown in Figure 7.

The presence of green polyhedra suggests the introduction of bulky groups, and the presence of blue polyhedra, more positive groups. This information is consistent with the docking studies performed to obtain the structural alignment. The best positioning of the molecules in the enzyme active site allows bulky groups to be added in these positions. When compared to the least active inhibitors, raltegravir has a bigger fragment A with more positive groups as oxadiazole ring and carboxamide group, both with nitrogen atoms. The presence of these groups is required for the interactions with the polar residues K156, K159 and T66 through hydrogen bonds. The presence of the red polyhedra in the chelating region indicates that high electron densities may increase the ligand affinities. Electronegative substituents in this position exert a greater effect on the affinity for Mg^2+^ ions. Possible molecular modifications can be performed to design more potent and more selective inhibitors, such as: (1) according to the electrostatic contour maps, a ketone group linked to pyrimidine ring can enhance the biological activity; (2) from the steric contour maps, a compound should have bulky substituents at the pyrimidone ring, for example, tetrahydrofuran, tetrahydropyran or morpholine.

### 4. HQSAR

From the patterns of fragment counts, several HQSAR models were generated and investigated. The statistical results from the PLS analyses for the 64 training set compounds, using several combinations of fragment distinctions, are presented in Table 2. From Table 2, we can observe that the best statistical results among all of the 13 generated models were obtained for model 5 (*q^2^* = 0.643 and *r^2^* = 0.908), which were derived using A/B/C/H/Ch/DA, respectively, and six being the optimum number of PLS components. This model was selected to investigate the influence of different fragment sizes on the main statistical parameters. The results obtained after the variation of the fragment sizes are displayed in Table 3. The best model generated was the one with fragment size equals 4–7, for the combination A/B/C/H/Ch/DA, with the best statistical results (*q^2^* = 0.643 and *r^2^* = 0.908). Therefore, the final HQSAR model generated with the training set has the good cross-validation correlation coefficient as an indication of model robustness.

**Table 2:** HQSAR analyses using several fragment distinctions with standard fragment size (4–7 atoms).

Model	Fragment Type	*q^2^*	SEP	*r^2^*	SEE	HL	N
**1**	A/B	0.482	0.461	0.737	0.329	353	3
**2**	A/B/C	0.464	0.469	0.722	0.388	353	3
**3**	A/B/C/H	0.510	0.461	0.894	0.214	257	6
**4**	A/B/C/H/Ch	0.547	0.443	0.857	0.233	97	6
**5**	**A/B/C/H/Ch/DA**	**0.643**	**0.349**	**0.908**	**0.199**	**61**	**6**
**6**	A/B/H	0.469	0.480	0.847	0.258	61	6
**7**	A/B/C/Ch	0.505	0.451	0.740	0.327	353	3
**8**	A/B/DA	0.527	0.449	0.823	0.274	83	5
**9**	A/B/C/DA	0.504	0.464	0.895	0.214	151	6
**10**	A/B/H/DA	0.561	0.433	0.834	0.266	83	5
**11**	A/B/C/Ch/DA	0.554	0.440	0.882	0.227	307	6
**12**	A/B/C/H/DA	0.516	0.450	0.752	0.322	71	4
**13**	A/B/H/Ch/DA	0.600	0.416	0.870	0.238	83	6

*q^2^: cross-validated correlation coefficient; SEP: cross-validated standard error; r^2^: correlation coefficient; SEE: standard error of estimation; HL: hologram length; N: optimal number of components. Fragment distinction: A, atoms; B, bonds; C, connections; H, hydrogen atoms; Ch, chirality; DA, donor and acceptor.*

**Table 3:** Influence of various fragment sizes for the model 6 with the combination A/B/C/H/Ch/DA.

Model	Fragment size	*q^2^*	SEP	*r^2^*	S	HL	N
**14**	**1–4**	0.390	0.514	0.842	0.262	401	6
**15**	**2–5**	0.506	0.463	0.895	0.214	151	6
**16**	**3–6**	0.563	0.435	0.896	0.212	401	6
**5**	**4–7**	**0.643**	**0.394**	**0.908**	**0.199**	**61**	**6**
**17**	**5–8**	0.559	0.433	0.855	0.248	199	5
**18**	**6–9**	0.586	0.426	0.916	0.190	307	6
**19**	**7–10**	0.577	0.428	0.912	0.196	151	6

**Figure 8:**
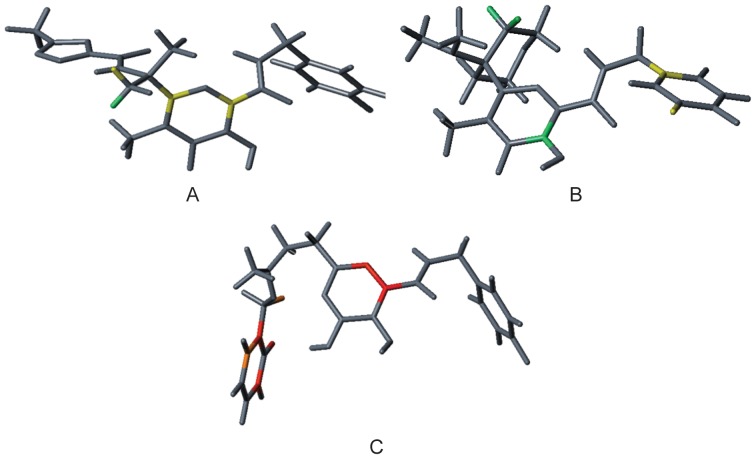
HQSAR contribution maps for raltegravir (A), the most (B) and the least (C) potent inhibitors. Fragments colored in green, blue-green and yellow contribute positively to the biological activity; fragments colored in red and orange contribute negatively to the biological activity.

The information contained in the best HQSAR model (model 5) is encoded in a contribution map, which shows every atom of a given molecule and its contribution to the biological activity. On this map, colors in the red region of the spectrum reflect unfavorable or negative contributions, and colors in the green region reflect favorable or positive contributions. Atoms with intermediate contributions are colored in white. Figure 8 shows the contribution maps for raltegravir (**12**), the most (**2**) and the least (**37**) potent inhibitors of the data set.

The contribution map for raltegravir and the most potent inhibitor (2), displayed in Figures 8A and 8B, shows that the hydrogen atom in the methyl group is suggested to be a favorable contribution for anti-HIV activity, in contrast to the phenolic ring in the least potent compound (37), which contributes negatively to the biological activity, suggesting that this fragment is a suitable target for molecular modification and further SAR studies.

### 5. External Validation

**Figure 9:**
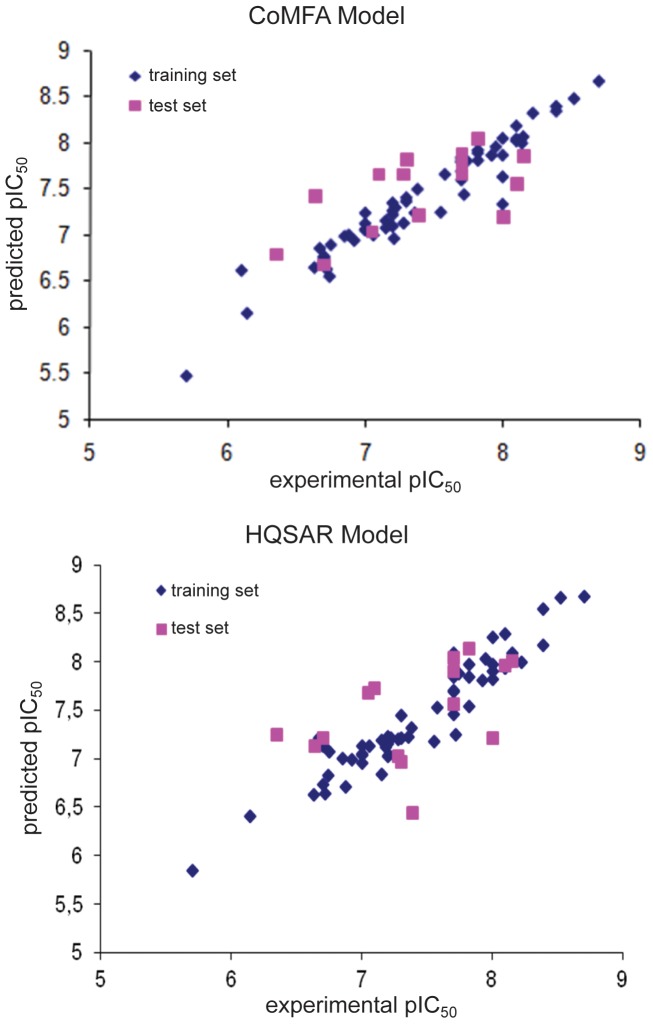
Predicted *versus* experimental pIC_50_ for training and test sets obtained from CoMFA and HQSAR models.

In order to ascertain the abilities of the obtained models in making predictions for unknown compounds, a test set chosen in such a way that it represents the chemical diversity and the distribution of biological activity, was employed to perform the external validation. Table 4 shows the results of the predictions for the CoMFA and HQSAR models for the test set, while graphical results for experimental versus predicted values are displayed in Figure 9.

**Table 4:** Experimental and predicted activities (pIC_50_), along with the residual values, for the test set using HQSAR and CoMFA methods.

Compound	pIC_50_ Experimental	CoMFA pIC_50_ Predicted	CoMFA Residual	HQSAR pIC_50_ Predicted	HQSAR Residual
**65**	6.64	7.43	−0.79	7.13	−0.49
**66**	7.82	8.04	−0.22	8.13	−0.31
**67**	7.70	7.87	−0.17	7.91	−0.21
**68**	7.70	7.81	−0.11	8.04	−0.34
**69**	8.15	7.86	0.29	8.01	0.14
**70**	7.28	7.65	−0.37	7.03	0.25
**71**	7.30	7.81	−0.51	6.97	0.33
**72**	8.00	7.19	0.81	7.21	0.79
**73**	7.39	7.21	0.18	6.45	0.94
**74**	7.05	7.03	0.02	7.68	−0.63
**75**	7.10	7.65	−0.55	7.72	−0.62
**76**	6.35	6.78	−0.43	7.25	−0.90
**77**	8.10	7.55	0.55	7.96	0.14
**78**	7.70	7.66	0.04	7.56	0.14
**79**	6.70	6.67	0.03	7.21	−0.51

The low differences between experimental and predicted values indicate that both models present a good predictive ability. The external validation process shows that these models are able to predict the biological activity of other molecules within this chemical class, represented by the diversity within the chemical space explored.

## Conclusions

In this study, we presented molecular docking and quantum chemical studies, along with CoMFA and HQSAR models, successfully applied to a series of HIV-1 IN inhibitors. By the analysis of molecular docking, it was possible to verify the binding modes of the inhibitors at the enzyme active site, which occur by chelation of the two Mg^2+^ ions. Additional interactions are observed in raltegravir drug and the most potent molecules, which show a better positioning of fragment A into one of the active site cavities, due to interactions with T66, K159 and K156 residues. The DFT results indicated that, for the most active molecules, the HOMO molecular orbitals are located mainly in the chelating domain. So, there is an important contribution of the negative charges on the oxygen atoms that are involved in coordination with the Mg^2+^ ions. This orbital effect produces a high nucleophilic character on these active sites. On the other hand, LUMO of the more active molecules is located in the Mg^2+^ region, enabling interactions with oxygen atoms of aspartate (D64 and D116) and glutamate (E152) residues in the enzyme active site. The resulting 3D QSAR model was able to provide a good correlation between the steric and electrostatic fields with the docking results. The contour maps indicated that in the chelating region, more electronegative groups are needed, and that in the fragment A, substitutions by more positive and bulkier groups contribute to interactions with polar T66, K159 and K156 residues. Additionally, information obtained from HQSAR contribution maps indicates the importance of substituents in fragment A to the design of new HIV-1 IN Inhibitors.

## Supporting Information

Table S1 **Chemical structures and IC_50_ values for the HIV-1 N inhibitors.**
(DOCX)
