# Supplementary material for: Molecular Features Related to HIV Integrase Inhibition Obtained from Structure- and Ligand-Based Approaches
Source: PLoS One. 2014 Jan 8;9(1):e81301. doi: 10.1371/journal.pone.0081301 (PMC3885377; doi:10.1371/journal.pone.0081301)
Supplement: Table S1 — Chemical structures and IC50 values for the HIV-1 N inhibitors. (DOCX) [file pone.0081301.s001.docx]

**Supporting Information**

**Table S1**. Chemical structures and IC_50_ values for the HIV-1 N inhibitors

| **Training set** | | | | |
| --- | --- | --- | --- | --- |
| **Compound** | **General Structure** | **R_1_** | **R_2_** | **pIC_50_** |
| **1** | 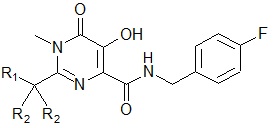 | 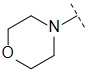 | Me | 8.5 |
| **2** |  | 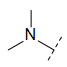 | 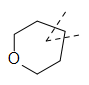 | 8.7 |
| **3** |  | 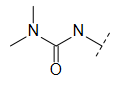 | Me | 7.7 |
| **4** |  | 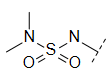 | Me | 7.9 |
| **5** | 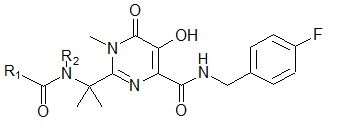 | 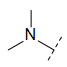 | H | 8.0 |
| **6** |  | 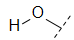 | H | 8.4 |
| **7** |  | 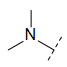 | Me | 7.8 |
| **8** | 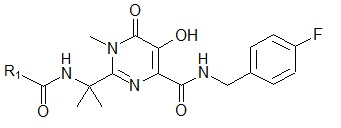 | 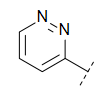 | - | 7.8 |
| **9** |  | 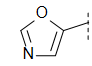 | - | 8.1 |
| **10** |  | 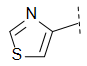 | - | 8.0 |
| **11** |  | 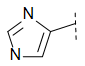 | - | 8.2 |
| **12** |  | 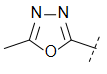 | - | 7.8 |
| **13** |  | 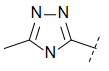 | - | 8.4 |

| **Cpd.** | **General Structure** | **R_1_** | **R_2_** | **pIC_50_** |
| --- | --- | --- | --- | --- |
| **14** | 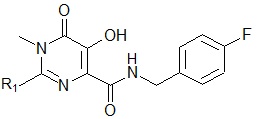 | 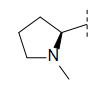 | - | 7.2 |
| **15** |  | 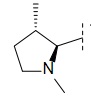 | - | 6.1 |
| **16** |  | 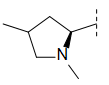 | - | 6.7 |
| **17** |  | 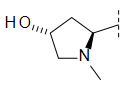 | - | 7.0 |
| **18** |  | 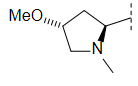 | - | 6.7 |
| **19** |  | 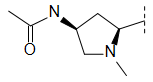 | - | 7.2 |
| **20** |  | 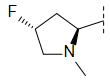 | - | 7.7 |
| **21** |  | 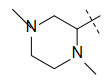 | - | 7.0 |
| **22** |  | 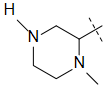 | - | 6.7 |
| **23** |  | 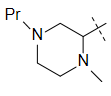 | - | 6.8 |
| **24** |  | 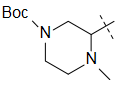 | - | 7.4 |

| **Cpd.** | **General Structure** | **R_1_** | **R_2_** | **pIC_50_** |
| --- | --- | --- | --- | --- |
| **25** | 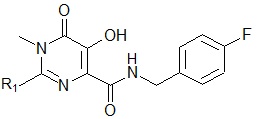 | 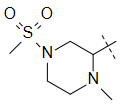 | - | 7.1 |
| **26** |  | 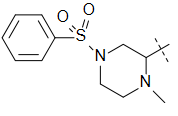 | - | 8.2 |
| **27** |  | 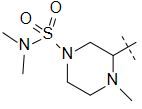 | - | 8.1 |
| **28** |  | 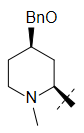 | - | 7.2 |
| **29** |  | 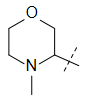 | - | 7.2 |
| **30** |  | 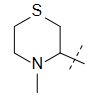 | - | 7.1 |
| **31** | 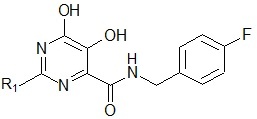 | 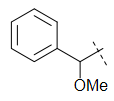 | - | 7.1 |
| **32** |  | 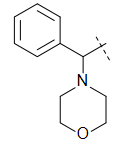 | - | 7.3 |
| **33** |  | 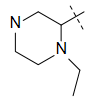 | - | 7.0 |
| **34** |  | 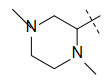 | - | 7.0 |
| **35** |  | 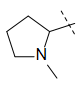 | - | 6.6 |
| **36** |  | 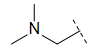 | - | 6.7 |

| **Cpd.** | **General Structure** | **R_1_** | | | **R_2_** | | **pIC_50_** |
| --- | --- | --- | --- | --- | --- | --- | --- |
| **37** | 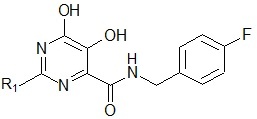 | 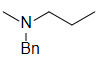 | | | - | | 5.7 |
| **38** |  | 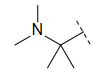 | | | - | | 7.3 |
| **39** |  | 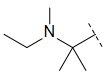 | | | - | | 7.2 |
| **40** |  | 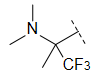 | | | - | | 8.1 |
| **41** |  | 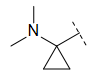 | | | - | | 8.0 |
| **42** |  | 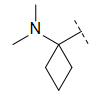 | | | - | | 7.3 |
| **43** |  | 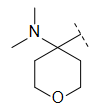 | | | - | | 7.5 |
| **44** |  | Pr | | | - | | 7.0 |
| **45** |  | H | | | - | | 7.2 |
| **46** | 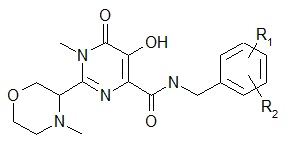 | H | | | H | | 7.2 |
| **47** |  | 2-F | | | H | | 6.7 |
| **48** |  | 3-F | | | H | | 7.2 |
| **49** |  | 3-Cl | | | H | | 7.2 |
| **50** |  | 3-Br | | | H | | 7.7 |
| **51** |  | 3-OMe | | | H | | 6.8 |
| **52** |  | 4-F | | | 3-F | | 7.6 |
| **53** |  | 4-F | | | 3-Me | | 7.1 |
| **54** |  | 4-F | | | 3-Cl | | 7.5 |
| **55** |  | 4-F | | | 3-Br | | 7.3 |
| **Cpd.** | **General Structure** | **R_1_** | | | **R_2_** | | **pIC_50_** |
| **56** | 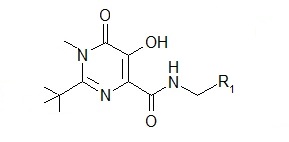 | 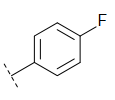 | | | - | | 8.0 |
| **57** |  | 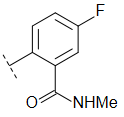 | | | - | | 7.7 |
| **58** |  | 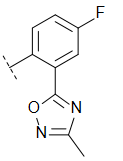 | | | - | | 7.7 |
| **59** | 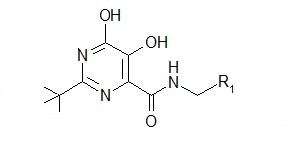 | 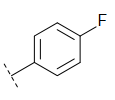 | | | - | | 8.0 |
| **60** |  | 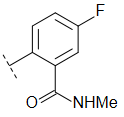 | | | - | | 7.7 |
| **61** |  | 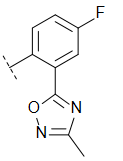 | | | - | | 7.7 |
| **62** |  | 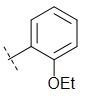 | | |  | | 8.0 |
| **63** |  | 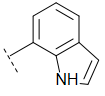 | | |  | | 7.2 |
| **64** |  | 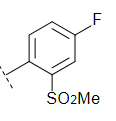 | | |  | | 8.0 |
| **Test set** | | | | | | | |
| **Cpd.** | **General Structure** | | **R_1_** | **R_2_** | | **pIC_50_** | |
| **65** | 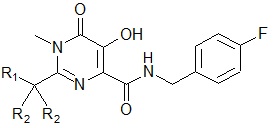 | 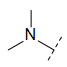 | | | Me | | 6.6 |
| **66** |  | 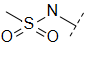 | | | Me | | 8.1 |
| **67** | 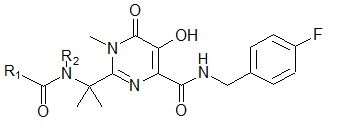 | 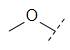 | | | H | | 7.8 |
| **68** |  | 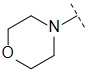 | | | H | | 7.7 |

| **69** | 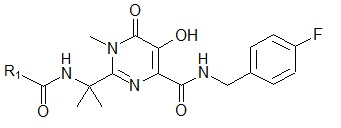 | 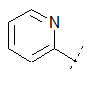 | - | 7.7 |
| --- | --- | --- | --- | --- |
| **70** |  | 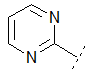 | - | 8.2 |
| **71** | 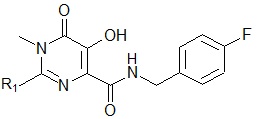 | 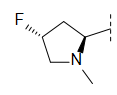 | - | 7.7 |
| **72** |  | 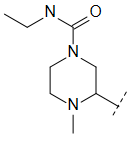 | - | 7.3 |
| **73** |  | 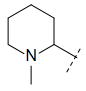 | - | 6.4 |
| **74** |  | 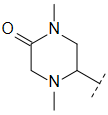 | - | 7.3 |
| **Cpd.** | **General Structure** | **R_1_** | **R_2_** | **pIC_50_** |
| **75** | 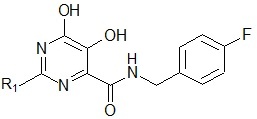 | 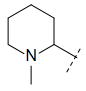 | - | 6.7 |
| **76** |  | 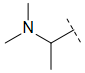 | - | 8.0 |
| **77** |  | 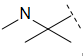 | - | 7.4 |
| **78** |  | 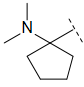 | - | 7.0 |
| **79** |  | 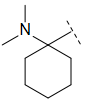 | - | 7.1 |
